# An algebraic language for RNA pseudoknots comparison

**DOI:** 10.1186/s12859-019-2689-5

**Published:** 2019-04-18

**Authors:** Michela Quadrini, Luca Tesei, Emanuela Merelli

**Affiliations:** School of Science and Technology, University of Camerino, Via Madonna della Carceri 9, Camerino, 62032 Italy

**Keywords:** Tree grammar, Tree alignment, Algebraic RNA tree, Structural RNA tree, ASPRA distance

## Abstract

**Background:**

RNA secondary structure comparison is a fundamental task for several studies, among which are RNA structure prediction and evolution. The comparison can currently be done efficiently only for pseudoknot-free structures due to their inherent tree representation.

**Results:**

In this work, we introduce an algebraic language to represent RNA secondary structures with arbitrary pseudoknots. Each structure is associated with a unique algebraic RNA tree that is derived from a tree grammar having *concatenation*, *nesting* and *crossing* as operators. From an algebraic RNA tree, an abstraction is defined in which the primary structure is neglected. The resulting structural RNA tree allows us to define a new measure of similarity calculated exploiting classical tree alignment.

**Conclusions:**

The tree grammar with its operators permit to uniquely represent any RNA secondary structure as a tree. Structural RNA trees allow us to perform comparison of RNA secondary structures with arbitrary pseudoknots without taking into account the primary structure.

## Background

RNA is a single stranded polymer, called primary structure, that consists of four different nucleotides - Adenine (A), Guanine (G), Cytosine (C) and Uracil (U) - linked together by phosphodiester bonds, referred to as strong bonds. RNA folds back on itself determining complex tree-dimensional shapes known as secondary and tertiary structures. During the folding process each nucleotide can interact with another one by establishing a hydrogen bond, referred to as weak bond, mainly forming Watson-Crick (G-C and A-U) and wobble (G-U) base pairs.

**Fig. 1:**
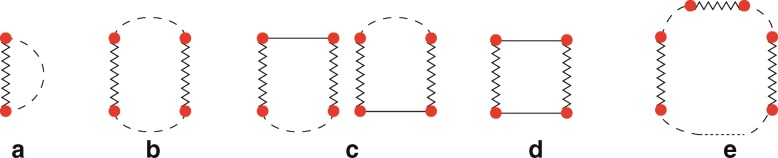
Basic structural elements of RNA secondary structures: **a**, hairpin; **b**, internal loop; **c**, bulge; **d**, helix and **e**, multi-loop. A strong interaction is depicted with a line, a weak interaction is drawn with a zigzagged line and several consecutive strong interactions are represented by a dashed line

According to Waterman [1, 2] RNA secondary structures can be decomposed into five basic structural elements, namely *hairpins*, *internal loops*, *bulges*, *helices* and *multi-loops*, as illustrated in Fig. 1. Each structural element is generated when at least one base pair is established and is characterised by strong and weak interactions determining a *loop*. A hairpin (Fig. 1a) is a loop characterised by one weak bond enclosing a sequence of nucleotides linked by strong bonds. An internal loop (Fig. 1b) is defined by two weak bonds alternating with two non-empty sequences of nucleotides linked by strong bonds. A bulge (Fig. 1c) is a special case of internal loop in which one of the two sequences of nucleotides is empty. A helix (Fig. 1d) is also a special case of internal loop in which both sequences are empty. Finally, a multi-loop (Fig. 1e) consists of more than two weak bonds separated by non-empty sequences of nucleotides linked by strong bonds.

**Fig. 2:**
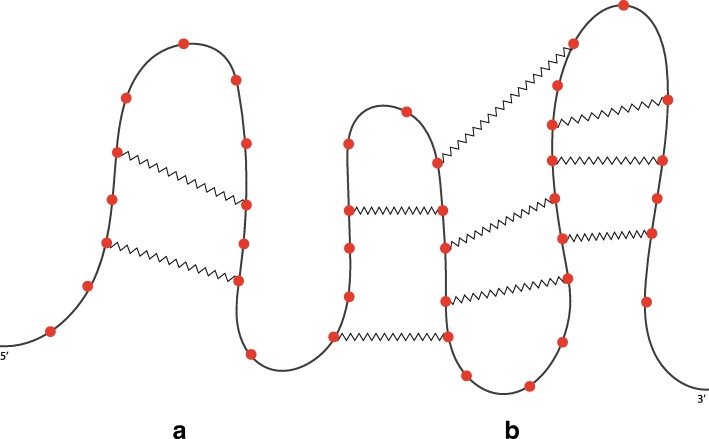
An RNA secondary structure. Each nucleotide is represented by a ball, a strong interaction is depicted by a line and a weak interaction by a zigzagged line. **a**, pseudoknot-free; **b**, a pseudoknot, which makes the whole structure pseudoknotted

**Fig. 3:**
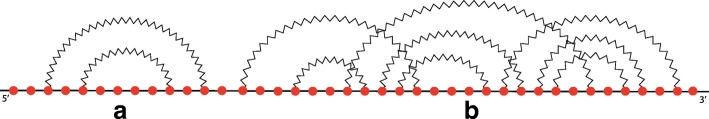
The secondary structure of Fig. 2. In **a**, the zigzagged arcs do not cross while, in **b**, pseudoknots are clearly visible as crossings of arcs

Disregarding the spatial configuration of the molecule and reducing nucleotides to dots, an RNA secondary structure can be schematically represented by a planar diagram like the one in Fig. 2, where solid and zigzagged lines represent strong and weak bonds, respectively. Each planar diagram can be transformed into another one (see Fig. 3) where the nucleotides are represented by vertices on a straight line (backbone) and the base pairs are drawn as arcs in the upper half-plane. Note that Fig. 3 shows the same molecule of Fig. 2. A secondary structure is said to be *pseudoknot-free* if the diagram does not present crossing among base pairs (Fig. 3a), otherwise it is called *pseudoknotted* (Fig. 3b).

Pseudoknots are very significant for the functional aspects of the RNA structures where they are present [3] and they are actually frequently found in real RNAs [4]. It is recognised that they play a variety of roles in biology, for example in the formation of the catalytic core of various ribozymes [5, 6] and in the alteration of gene expression inducing ribosomal frameshifting in many viruses [7, 8].

The ability to compare RNA structures is useful for the prediction of the RNA folding process taking as initial data a set of already known secondary structures [9]. It is also useful for the RNA classification of various species [10, 11], for determining the RNA consensus structure of aligned sequences and for the identification of highly conserved structures during evolution [12, 13]. Functional RNA families such as tRNA, rRNA, and RNAse P exhibit a highly conserved shape of secondary structure but little sequence similarity [14]. Therefore, it is of great interest the possibility of comparing RNA secondary structures directly, i.e., without relying on sequence similarity [15–17].

Many approaches for pseudoknot-free RNA secondary structure comparison are based on their natural context-free tree representation. Thus, the comparison of pseudoknot-free structures can be reduced to *tree comparison*. Tree comparison was firstly introduced by Selkow in 1977. He gave an algorithm that transforms a given tree into another one by performing a sequence of *edit operations* (namely, deletion, insertion and replacement of nodes) with minimal score [18]. Selkow’s approach had the limitation that the edit operations could be applied only to the leaf nodes of the trees. Later, Tai’s work used the same edit operations but permitted their application to all nodes [19]. Another comparison technique is based on *tree alignment*, defined by Jiang et al. [20]. Given two trees, it constructs an alignment tree in which they can be embedded homomorphically. Tree editing and tree alignment are both based on edit operations and minimise a score function associated with them. This minimal score is usually referred to as *distance* and is used as a measure of similarity among structures. Höchsmann et al. extended the tree alignment algorithm to compute the local alignment of forests and, based on the forest alignment model, developed a multiple alignment algorithm. *RNAforester* is the software package that implements these algorithms for pseudoknot-free RNA secondary structure comparison [14, 21]. RNAforester is distributed within the ViennaRNA package [22]. Chauve et al. defined the first unambiguous and complete dynamic programming for tree alignment [23]. Many other authors contributed in this field although the computational complexity has not been improved [24]. For a complete treatment of tree editing and tree alignment we refer to Bille’s survey [25]. In the RNA setting, the tree editing approach is useful to identify the conserved structures during the folding process, while the tree alignment is suitable for clustering RNA molecules purely at the *structural level*.

Although motifs with pseudoknots are considered important, most of the comparison approaches in the literature exclude pseudoknotted structures. One of the main reasons for this lack is the fact that the classical tree representation of structures fails when pseudoknots are present [26]. However, in the literature there are some works in which pseudoknotted structures are represented with other kinds of mathematical structures. Möhl et al. proposed a sequence-structure alignment for RNA pseudoknots which involves a pipeline for combining alignment and prediction of pseudoknots [27]. Han et al. decomposed embedded pseudoknots into simple pseudoknots and aligned them recursively [28]. Yoon used a profile hidden Markov model to establish sequence alignment constraints and incorporated these constraints into an algorithm for aligning RNAs with pseudoknots [29]. Wong et al. identified the pseudoknot type of a given structure and developed dynamic programming algorithms for structural alignments of different pseudoknot types [30]. Huang et al. applied a tree decomposition algorithm to search for non-coding RNA pseudoknotted structures in genomes [31].

Parallel to structure comparison, structure prediction is one of the most extensively studied problems about RNA secondary structures. For the state of the art about folding algorithms we refer to a recent survey [32]. Regarding the folding of pseudoknotted structures, efficient algorithms exist only for particular classes of pseudoknots because finding the best structure including arbitrary pseudoknots is an NP-complete problem [33]. An overview of the various classes of pseudoknots is given by Nebel and Weinberg [34]. In particular, Reeder and Giegerich introduced the class of canonical simple recursive pseudoknots and developed an algorithm for folding structures possibly containing these motifs [35]. Notably, they used the general framework of *Algebraic Dynamic Programming* (ADP) [36, 37], which has been recently extended by Berkemer et al. to tree grammars and applied to tree alignment and tree editing [38]. Riechert et al. expanded ADP to be used with multiple context-free grammars and applied the method to the classes of RNA pseudoknotted secondary structures that can be expressed by multiple context-free grammars [39]. Ponty and Saule unified in the same framework the dynamic programming algorithms for the folding of several classes of RNA secondary structures with pseudoknots based on hypergraph representation [40].

In this paper we tackle the problem of structural comparison of RNA secondary structures with arbitrary pseudoknots based on an algebraic language for their representation as trees. We reuse the existing familiar notion of tree alignment with the relative optimised algorithms. A distance is defined among structures that neglects the primary structure and focuses on weak interactions.

**Fig. 4:**
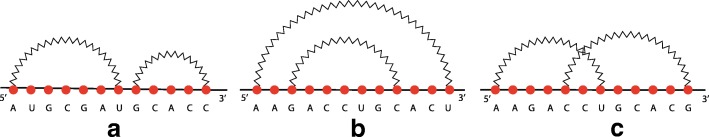
**a**, concatenation **b**, nesting and **c**, crossing of two hairpins

We introduce first a set of appropriate operators, namely *concatenation*, *nesting* and *crossing*, which are defined to express each RNA secondary structure as an algebraic composition of hairpin loops. Briefly, concatenation is used to represent a motif in which a structure is followed by another one, as illustrated in Fig. 4a for two simple hairpins. Nesting corresponds to the insertion of a structure into a hairpin (Fig. 4b, where the internal structure is a simple hairpin) and crossing models interaction among structures (Fig. 4c, where both structures are simple hairpins). According to the nature of RNA molecules, nesting and crossing are well defined if each nucleotide of the resulting structure forms at most one base pair. Such constraints do not apply to concatenation because two concatenated structures do not share nucleotides.

Using the defined operators, we introduce a *regular tree grammar* with conditional productions to obtain a *unique* tree representation of each RNA secondary structure, both pseudoknot-free and pseudoknotted. The use of tree grammars is inspired by the ADP framework, which is mainly used in the context of folding. In this work we use only the tree language part of the ADP framework because we focus on the representation of structures. As future work, we plan to use the full framework for exploiting our algebraic operators in the context of folding.

The derived trees of the defined regular tree grammar are called *algebraic RNA trees* and they are shown to be in a one-to-one correspondence with RNA secondary structures. This representation emphasises the algebraic nature of our approach, but contains too much information for the purpose of structural comparison by tree alignment. Therefore, we abstract them and we derive *structural RNA trees* by forgetting the primary structure. The algebraic aspect will be investigated in a future work to obtain a formalisation of a real algebra similar to Allen’s Interval Algebra [41]. Our *distance* is calculated by aligning structural RNA trees using a scoring function that takes into account deletion, insertion and replacement of operators and hairpins, together with the number of crossings among hairpins.

We implemented the construction of algebraic and structural RNA trees together with the alignment and the calculation of the distance in the ASPRAlign open source Java application [42] that is distributed under the GNU General Public Licence, version 3.

## Methods

### Regular tree grammars

The theory of tree automata and tree languages was introduced in the middle of the 1960s by Thatcher [43] and extended in the following years [44]. Let us give a brief presentation of regular tree grammars in the style used within the ADP framework [36, 37].

#### **Definition 1**

(Signature) Let $\mathcal {A}$ be an alphabet of symbols. A *signature* *Σ* over $\mathcal {A}$ consists of:

- a name for a base set, say *D*, intended as a placeholder for a yet unspecified data domain;
- a family of function names (operators), together with their argument and result types, where
  - argument types are either *D* or $\mathcal {A}$, and
  - the result type is always *D*.

A signature contains the building blocks for constructing terms, which are all the well-typed formulas that can be formed using the symbols in $\mathcal {A}$ and the function names of the signature *Σ*. Each term can be naturally viewed as a rooted, ordered, labeled tree; actually, in this context, they can be identified, thus we will speak equivalently of terms or trees. The set containing all terms is denoted by *T*_*Σ*_ and is the analogous of the universe set $\mathcal {A}^{*}$ for string languages. As it happens for strings, usually it is convenient to consider only a subset of all possible trees, which leads to the definition of tree languages.

#### **Definition 2**

(Tree Language) Let $\mathcal {A}$ be an alphabet and let *Σ* be a signature. A tree language defined by *Σ* over $\mathcal {A}$ is any subset of *T*_*Σ*_.

Along the analogy with string languages, a tree language can be defined by a tree grammar. Among different types of tree grammars, having different expressive powers, we will use *regular* tree grammars. For convenience we permit variables, used as non-terminal symbols, in terms. If *V* is a set of variables then *T*_*Σ*_(*V*) denotes the set of all the terms in which a variable in *V* can occur as a term or in place of a sub-term.

#### **Definition 3**

(Regular Tree Grammar) A regular tree grammar $\mathcal {G}$ over *Σ* is a tuple $(V,\Sigma,S,\mathcal {P})$ where:

- *V* is a set of non-terminal symbols;
- *Σ* is a signature;
- *S*∈*V* is a designed non-terminal symbol called axiom;
- $\mathcal {P}$ is a set of productions of the form *v*→*t*, where *v*∈*V* and *t*∈*T*_*Σ*_(*V*).

A derivation relation for regular tree grammars $\xrightarrow {*}$ is defined analogously to the one of context-free grammars. Starting from the axiom, each non-terminal symbol *v* can be rewritten with a tree *t* whenever *v*→*t* belongs to $\mathcal {P}$.

#### **Definition 4**

(Language of a Regular Tree Grammar) Let $\mathcal {G} = (V,\Sigma,S,\mathcal {P})$ be a regular tree grammar. The tree language generated by $\mathcal {G}$ is

$$\mathcal{L}(\mathcal{G}) = \{ t \in T_{\Sigma} \mid S \xrightarrow{*} t\}$$

As an example, let $\mathcal {A} = \{a,b,c,d,e\}$ be an alphabet and let *Σ*={*p,q*}, with $p\colon \mathcal {A} \times D \times \mathcal {A} \rightarrow D$ and $q\colon D \times \mathcal {A} \rightarrow D$, be a signature over $\mathcal {A}$. Let $\mathcal {G}_{1} = (\{S,T\}, \Sigma, S, P)$ be a regular tree grammar where $\mathcal {P}$ consists of the rules:

$$\begin{array}{lll} S & \to & p(a, T, a) \\ T & \to & q(p(c, T, d), b) \mid e \end{array} $$

Then, for instance, $\mathcal {G}_{1}$ generates the term *p*(*a,q*(*p*(*c,e*,*d*),*b*),*a*) as follows:

$$\begin{aligned} S \rightarrow p(a, T, a) \rightarrow p(a, q(p(c, T, d), b), a) \rightarrow p(a, q(p(c, e, d), b), a) \end{aligned} $$

or, pictorially, as trees:


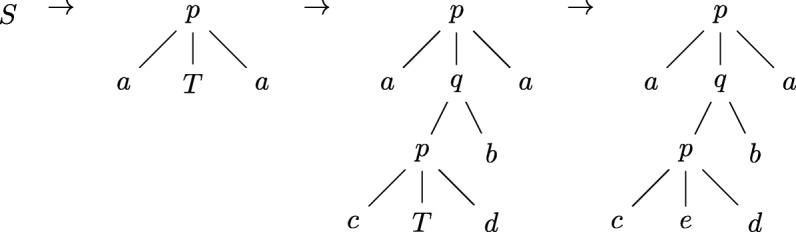


In the literature, each tree obtained with the tree grammar is known as *derived tree*. For each derived tree, its *yield* is normally defined as the sequence of leaf symbols read in left to right order. Formally, the *yield functiony* on a tree *t*∈*T*_*Σ*_ is defined as

$$y(t) = \left \{ \begin{array}{lll} a & \text{ if} & t = a \text{ is a leaf }\\ y(t_{1}) \cdot y(t_{2}) \cdot \, \cdots \, \cdot y(t_{n}) & \text{ if} & t = f(t_{1},t_{2},\ldots,t_{n}) \end{array} \right. $$

thus, for each tree *t*, $y(t) \in \mathcal {A}^{*}$. In the example above, the yield of the derived tree *p*(*a,q*(*p*(*c,e*,*d*),*b*),*a*) is the string *acedba*.

For brevity, as proposed for instance in [45], we add a *lexical level* to the grammar, i.e., we allow strings from $\mathcal {A}^{*}$ to label the leaf of trees, instead of constraining to a single symbol. Moreover, for the purposes of this paper, we will also use *conditional productions*, i.e. syntactic conditions associated with productions, as defined in the following.

#### **Definition 5**

(Conditional Production) In a regular tree grammar, a conditional production has the form $ v \xrightarrow {c} t $ where *c* is a predicate defined on $\mathcal {A}^{*}$. A derivation using a conditional production $v \xrightarrow {c} t$ is well formed if and only if the tree *t*^′^∈*T*_*Σ*_ obtained from *t* at the end of the derivation, i.e. $t \xrightarrow {*} t'$, is such that *c*(*y*(*t*^′^)) holds.

The language of a regular tree grammar with conditions is the set of trees that can be derived from the axiom only by well-formed derivations.

Note that the use of a conditional production $v \xrightarrow {c} t$ at some point in a derivation affects the complete derivation that continues from the subtree *t* inserted by this production. Only after the derivation is complete the condition can be checked.

### Tree alignment

Tree alignment is a generalisation of sequence alignment. Let us introduce the notion of tree alignment by analogy with that of sequence alignment. An alignment of two sequences of characters can be seen as a sequence of character pairs, where pairs of type (*a,b*) are *replacements*, (*a*,−) are *deletions* and (−,*a*) are *insertions*. Note that *a,b* are alphabet characters of the sequence, whereas “-”, referred to as the *gap symbol*, is not an element of the alphabet. Let *s*_1_ and *s*_2_ be two sequences over an alphabet and let *s*_1_[ *i*] (*s*_2_[ *i*]) denote the *i*-th element of the sequence *s*_1_ (*s*_2_). An alignment of *s*_1_ and *s*_2_ is denoted *s*^′^=(*s*1′,*s*2′) and is such that each element of a pair (*s*1′[ *i*], *s*2′[ *i*]) may be the gap symbol, but the pair (−,−) is not allowed. The *score* of an alignment of two sequences is given by

$$\sigma(s') = \sum\limits_{i=1}^{l} \sigma\left(s'_{1}[\!i], s'_{2}[\!i]\right) $$

where *σ* is a scoring function such that *σ*(*x,y*)=0 if *x*=*y*≠− and *σ*(*x,y*)=1 otherwise. With an abuse of notation, here and in the following definition *σ* is used both for the scoring function and the score. An *optimal alignment* is an alignment with the minimum score.

The alignment of trees can be defined by analogy. An alignment of two ordered labelled trees is a tree whose nodes carry pairs that represent deletions, insertions, and replacements as defined for the alignment of sequences. Given two trees *t*_1_ and *t*_2_, to obtain an alignment tree they must first be modified by inserting nodes labelled with the gap symbol in such a way that they become isomorphic. Then, the two isomorphic trees are overlaid forming only one tree *L* in which each node contains the pair of the labels coming from the two isomorphic trees.

#### **Definition 6**

(Tree Alignment Distance) Let *t*_1_ and *t*_2_ be two trees. The tree alignment distance between *t*_1_ and *t*_2_, denoted by *d*_*T*_(*t*_1_,*t*_2_), is the minimum score over all possible alignments of the two trees:

$$d_{T}(t_{1}, t_{2}) = \min{ \{\sigma(L) \mid L \text{ is an alignment of}\ t_{1} \text{ and}\ t_{2} \} } $$

where $\sigma (L)= \sum _{(a,b) \in L} \sigma (a,b)$ and *σ* is a scoring function.

The tree alignment distance is not a metric, as it does not satisfy the triangle inequality. The tree alignment problem, i.e. finding the alignment with the optimal score, can be solved by considering all possible candidate alignments in a dynamic programming algorithm. A classical tree alignment algorithm was proposed by Jiang et al. in [20]. For a complete treatment of tree alignment we refer to the tutorial of Schirmer et al. [46].

## Results

### Algebraic operators for RNA secondary structures

As introduced in the “Background” section, Waterman showed that each pseudoknot-free RNA secondary structure can be uniquely decomposed into five basic structural elements, or loops [1, 2]. Among these loops, hairpin is the basic one, consisting of only one weak interaction closing a sequence of unpaired nucleotides. Inspired by the Waterman’s result, our first objective is to define a set of algebraic operators able to represent *any kind* of RNA secondary structure, including the pseudoknotted ones, as a combination of simple hairpin loops. In other words, our aim is to express any RNA secondary structure as an *algebraic expression* that, using only the defined operators, combines simple hairpins in a proper way to obtain a unique representation of the structure.

**Fig. 5:**
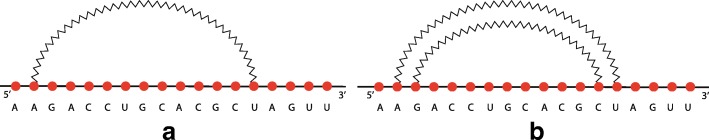
**a**, an hairpin and **b**, the nesting of two hairpins

**Fig. 6:**
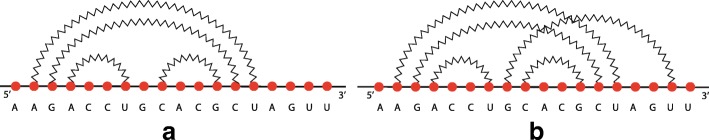
**a**, concatenation of two hairpins nested into two nested hairpins. **b**, pseudoknot created by adding a new weak interaction between an unpaired nucleotide of the structure **a** and one belonging to the tail

Let us first introduce informally all the needed ingredients by using some examples. First, we observe that, starting from a primary structure, the introduction of a weak interaction creates a secondary structure, which is composed of a *head* sequence, followed by a hairpin loop, followed by another sequence of unpaired nucleotides, which is a *tail*. Figure 5a shows an example of this case, where the head is formed only by the nucleotide *A* and the tail is the sequence *AGUU*. Secondly, we observe that the introduction of a new weak interaction between two unpaired nucleotides inside an hairpin generates another loop that is *nested* into the other, as illustrated in Fig. 5b. *Nesting* is the first of our operators, used to represent these situations. Thirdly, we observe that adding simultaneously two or more new weak interactions that involve nucleotides of an existing loop, in such a way that they do not cross, the result is the appearing of two or more new hairpins. They are *concatenated* and linked by a possibly empty sequence of unpaired nucleotides. Figure 6a shows this case by introducing into the structure of Fig. 5b two new weak interactions. The two new hairpins are linked by the nucleotide *G*. *Concatenation* is then another operator, introduced to model these situations. Finally, we observe that adding a new weak interaction involving two unpaired nucleotides of two different loops or one nucleotide of a loop and a nucleotide of the tail (or of the head), a pseudoknot is created. Figure 6b shows an example of the second case. The first nucleotide that belongs to the loop is connected with another one that composes the tail of the structure. Such weak interaction crosses with other weak interactions, calling for a *crossing* operator, which is the third of our set.

**Fig. 7:**
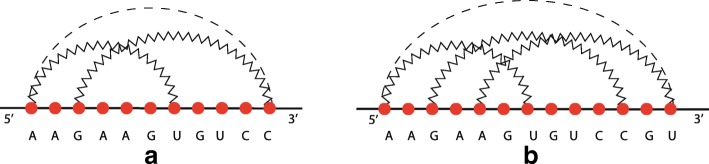
Two examples of pseudoloops. **a**, characterised by the crossing of two hairpins; **b**, characterised by the crossing of three hairpins

**Fig. 8:**
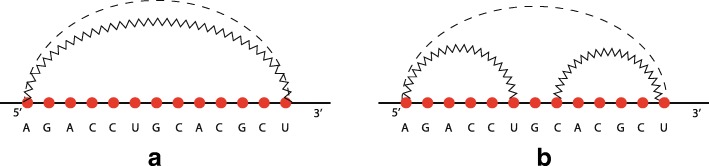
Two examples of pseudoloops without crossings. **a**, a hairpin; **b**, a concatenation of two hairpins

To define the three operators more formally we need to introduce the concept of *pseudoloop*, a structure characterised by zero or more crossings of weak interactions. Two examples of pseudoloops are illustrated in Fig. 7: a structure with one crossing and another one with three crossings. Graphically, we identify a pseudoloop with a dashed line and call it *pseudoweak interaction*, a fictitious weak interaction that links the first and the last nucleotide of the structure. Note that pseudoloops are just secondary structures without heads and tails. Note also that a simple hairpin loop or a concatenation of hairpin loops is a pseudoloop, in this case having no crossings. Figure 8 illustrates these cases by showing two pseudoloops that are elements of the structure of Fig. 6b.

Formally, each pseudoloop will be denoted by an expression of the form $\left (a^{\bullet }_{1},a^{\bullet }_{N}\right) \langle \alpha \rangle $, where *α* is the sequence of nucleotides (backbone) enclosed by the pseudoweak interaction between the first nucleotide, $a^{\bullet }_{1}$, and the last one, $a^{\bullet }_{N}$. The ∙ notation indicates that the nucleotide is already paired with another one in a weak interaction. Conversely, the notation ∘ indicates that the nucleotide is unpaired. The sequence *α* may contain both paired and unpaired nucleotides. We will use the following convention throughout the paper: whenever we write an expression of the form *a*^*s*^ or $a^{s}_{i}$, where *a,a*_*i*_∈{*A,U*,*G,C*} and *s*∈{∘,∙}, we mean that *a* or *a*_*i*_ is the kind of nucleotide and *s* is the boolean information about its state of being paired or unpaired. Thus, if we write *a*_*i*_=*b*_*j*_ we mean that *a*_*i*_ and *b*_*j*_ are the same nucleotide, but we do not care about the pairing information. Instead, if we write an expression of the form *ais*_*i*_=*bjt*_*j*_, we impose that nucleotides *a*_*i*_ and *b*_*j*_ are the same and their states about pairing is the same as well. Finally, the pairing state notation may be omitted in contexts in which it can be neglected.

Our three operators are defined over pseudoloops: they take two pseudoloops and map them into another one. Note that a pseudoloop “forgets” the information about the actual weak interactions that exist inside it, retaining only the information about the pairing of nucleotides. This will not be a problem for our purposes because later we are going to use this concept just to check that the result of the application of the operators is well defined, which will not depend on the actual weak interactions that are inside the involved pseudoloops. Let us start defining the concatenation operator that takes two pseudoloops and attach them by a sequence of unpaired nucleotides.

#### **Definition 7**

(Concatenation) Let $P_{1} = \left (a^{\bullet }_{1},a^{\bullet }_{N}\right) \langle a_{2}\dots a_{N-1}\rangle $ and $P_{2} = \left (b^{\bullet }_{1},b^{\bullet }_{M}\right)\langle b_{2}\dots b_{M-1}\rangle $ be two pseudoloops such that *a*_*i*_,*b*_*j*_ are paired or unpaired nucleotides for all *i*=2,…,*N*−1 and *j*=2,…,*M*−1. Let *η*=*c*°_1_…*c*°_*T*_ be a possibly empty sequence of unpaired nucleotides. The *concatenation* of *P*_1_ and *P*_2_, denoted by *P*_1_⊙_*η*_*P*_2_, is defined as

$$P_{1}\odot_{\eta} P_{2} = \left(a^{\bullet}_{1},b^{\bullet}_{M}\right) \langle\ a_{2} \! \dots \! a_{N-1} a^{\bullet}_{N} \, \eta \, b^{\bullet}_{1} \dots b_{M-1} \rangle $$

As an example, consider the structure in Fig. 8b. This pseudoloop can be obtained as the concatenation of two pseudoloops *P*_1_=(*A*^∙^,*U*^∙^)〈*C*°*C*°〉 and *P*_2_=(*C*^∙^,*G*^∙^)〈*A*°*C*°〉 linked by a sequence composed of only one unpaired nucleotide, *G*. Thus,

$$P_{1} \odot_{G^{\circ}} P_{2} = (A^{\bullet},G^{\bullet}) \langle C^{\circ} C^{\circ} U^{\bullet} G^{\circ} C^{\bullet} A^{\circ} C^{\circ} \rangle$$

Differently from concatenation, the definition of the crossing of two pseudoloops is subject to constraints. In particular, it is necessary that a proper postfix of the primary sequence of the left pseudoloop is in common with a proper prefix of the primary sequence of the right one. Moreover, when the two pseudoloops are overlapped, the shared nucleotides must still retain the biological property that they are involved in at most one weak interaction. Thus, the definition that we are going to give necessarily introduces a notion of being well-defined, i.e., there may be crossings between pseudoloops that can not be considered valid in our setting. The definition also introduces a further parameter, a number *k* that holds the information about the position inside the left pseudoloop at which the right pseudoloop is attached (actually partially overlapped).

#### **Definition 8**

(Crossing) Let $P_{1} = \left (a^{\bullet }_{1},a^{\bullet }_{N}\right)\left \langle a^{s_{2}}_{2} \! \dots \! a^{s_{N-1}}_{N-1}\right \rangle $ and $P_{2} = \left (b^{\bullet }_{1},b^{\bullet }_{M}\right)\left \langle b^{t_{2}}_{2} \! \dots \! b^{t_{M-1}}_{M-1}\right \rangle $ be two pseudoloops such that *s*_*i*_,*t*_*j*_∈{∙,∘} for all *i*=2,…,*N*−1 and *j*=2,…,*M*−1. Let *k*∈{2,…,*N*−1} be an internal position of a nucleotide in *P*_1_. The *crossing* between *P*_1_ and *P*_2_, denoted by *P*_1_ ⋊⋉_*k*_*P*_2_, is defined as

$$P_{1} \Join_{k} P_{2} = \left(a^{\bullet}_{1}, b^{\bullet}_{M}\right) \left\langle \ a^{w_{2}}_{2} \! \dots \! a^{w_{k-1}}_{k-1} b^{w_{k}}_{1} \dots b^{w_{M+k-1}}_{M-1} \right\rangle $$

where

1. *M*+*k*−1>*N*, i.e., if *P*_2_ is attached starting from position *k* of *P*_1_, then the sequence of nucleotides of *P*_2_ ends after *P*_1_;
2. *b*_1_=*a*_*k*_,*b*_2_=*a*_*k*+1_,…,*b*_*M*−*k*_=*a*_*N*_, i.e., *P*_2_ shares with *P*_1_ the nucleotides from the *k*-th of *P*_1_ to the last one of *P*_1_; and
3. for all *z*=2,…,*M*+*k*−1
  $${\begin{aligned} w_{z} = \left\{ \begin{array}{lll} s_{z} & \text{ if} & z < k \\ t_{z} & \text{ if} & z > N \\ \circ & \text{ if} & k \leq z \leq N \, \wedge \, (s_{z} = \circ \, \wedge \, t_{z-k+1} = \circ)\\ \bullet & \text{ if} & k \leq z \leq N \, \wedge \, ((s_{z} = \bullet \, \wedge \, t_{z-k+1} = \circ) \, \vee \, (s_{z} = \circ \, \wedge \, t_{z-k+1} = \bullet))\\ \perp & \text{ if} & k \leq z \leq N \, \wedge \, (s_{z} = \bullet \, \wedge \, t_{z-k+1} = \bullet) \\ \end{array} \right. \end{aligned}} $$
  that is to say, each nucleotide in position *z* that is in common between *P*_1_ and *P*_2_ is involved in at most one weak interaction whenever *w*_*z*_≠⊥.

We say that the crossing *P*_1_ ⋊⋉_*k*_*P*_2_ is **well defined** if and only if conditions (1) and (2) above are met and for all *z*=2,…,*M*+*k*−1 it holds that *w*_*z*_≠⊥.

As an example, consider the structure in the Fig. 6b. The pseudoloop involving the nucleotides between the second (*A*^∙^) and the next to last (*U*^∙^) can be obtained as the crossing of *P*_1_=(*A*^∙^,*U*^∙^)〈*G*^∙^*A*^∙^*C*°*C*°*U*^∙^*G*°*C*^∙^*A*°*C*°*G*^∙^*C*^∙^〉 and *P*_2_=(*G*^∙^,*U*^∙^)〈*C*°*A*°*C*°*G*°*C*°*U*°*A*°*G*°〉, where *k* is equal to 7. Note that *P*_1_ corresponds to the structure of Fig. 6a. The result is

$$P_{1} \Join_{7} P_{2} \,=\, \!(A^{\bullet},U^{\bullet}) \!\langle \!G^{\bullet} A^{\bullet} C^{\circ} \!C^{\circ} U^{\bullet} G^{\bullet} C^{\bullet} A^{\circ} C^{\circ} G^{\bullet} C^{\bullet} U^{\bullet} A^{\circ} \!G^{\circ} \rangle$$

The nesting operator shares with the crossing one the fact that it is necessary to introduce a notion of being well-defined. However, for the nesting the constraints are slightly different. In particular, the nucleotides of the pseudoloop that is going to be nested inside the other one must all be shared, that is to say, the primary sequence of the nested pseudoloop must be a proper substring of the outer pseudoloop. The biological constraint about the weak interactions is the same. For convenience we impose that the outer pseudoloop of the nesting is the right operand of the operator while the nested pseudoloop is the left one. This decision influences also the position information held by the parameter *k*: in this case *k* is the relative position inside the outer (right) pseudoloop at which the nested (left) pseudoloop is overlapped.

#### **Definition 9**

(Nesting) Let $P_{1} = \left (a^{\bullet }_{1},a^{\bullet }_{N}\right)\left \langle a^{s_{2}}_{2} \! \dots \! a^{s_{N-1}}_{N-1}\right \rangle $ and $P_{2} = \left (b^{\bullet }_{1},b^{\bullet }_{M}\right)\left \langle b^{t_{2}}_{2} \! \dots \! b^{t_{M-1}}_{M-1}\right \rangle $ be two pseudoloops such that *s*_*i*_,*t*_*j*_∈{∙,∘} for all *i*=2,…,*N*−1 and *j*=2,…,*M*−1. Let *k*∈{2,…,*M*−2} be an internal position of a nucleotide in *P*_2_. The *nesting* of *P*_1_ and *P*_2_, denoted by $P_{1} \Cap _{k}P_{2}$, is defined as

$${\begin{aligned} P_{1} \Cap_{k} P_{2} = \left(b^{\bullet}_{1}, b^{\bullet}_{M}\right) \left\langle\ b^{w_{2}}_{2} \! \dots b^{w_{k-1}}_{k-1} a^{w_{k}}_{1} \dots a^{w_{N+k-1}}_{N} b^{w_{N+k}}_{N+k} \dots b^{w_{M-1}}_{M-1} \right\rangle \end{aligned}} $$

where:

1. *k*+*N*−1<*M*, i.e., *P*_1_ can be fully embedded inside *P*_2_ starting from position *k* of *P*_2_;
2. *a*_1_=*b*_*k*_,*a*_2_=*b*_*k*+1_,…,*a*_*N*_=*b*_*k*+*N*−1_, i.e., all nucleotides of *P*_1_ are shared with *P*_2_; and
3. for all *z*=2,…,*M*−1
  $${\begin{aligned} w_{z} = \left\{ \begin{array}{lll} t_{z} & \text{ if} & z < k \, \vee \, z > k + N - 1\\ \circ & \text{ if} & k \leq z \leq k + N -1 \, \wedge \, (t_{z} = \circ \, \wedge \, s_{z-k+1} = \circ)\\ \bullet & \text{ if} & k \leq z \leq k + N - 1 \; \, \wedge \\ & & ((t_{z} = \bullet \, \wedge \, s_{z-k+1} = \circ) \, \vee \, (t_{z} = \circ \, \wedge \, s_{z-k+1} = \bullet))\\ \perp & \text{ if} & k \leq z \leq k + N - 1 \, \wedge \, (t_{z} = \bullet \, \wedge \, s_{z-k+1} = \bullet) \\ \end{array} \right. \end{aligned}} $$
  that is to say, each nucleotide in position *z* that is in common between *P*_1_ and *P*_2_ is involved in at most one weak interaction whenever *w*_*z*_≠⊥.

We say that the nesting $P_{1} \Cap _{k} P_{2}$ is **well defined** if and only if conditions (1) and (2) above are met and for all *z*=2,…,*M*−1 it holds that *w*_*z*_≠⊥.

As an example of a well-defined nesting, consider the structure in Fig. 5b. The pseudoloop between the second and the fourteenth nucleotide can be obtained as the nesting of pseudoloops *P*_1_=(*G*^∙^,*C*^∙^)〈*A*°*C*°*C*°*U*°*G*°*C*°*A*°*C*°*G*°〉 and *P*_2_=(*A*^∙^,*U*^∙^)〈*G*°*A*°*C*°*C*°*U*°*G*°*C*°*A*°*C*°*G*°*C*°〉, with *k* equal to 2. The resulting pseudoloop is

$$P_{1} \Cap_{2} P_{2} = (A^{\bullet},U^{\bullet})\langle G^{\bullet} A^{\circ} C^{\circ} C^{\circ} U^{\circ} G^{\circ} C^{\circ} A^{\circ} C^{\circ} G^{\circ} C^{\bullet} \rangle $$

**Fig. 9:**
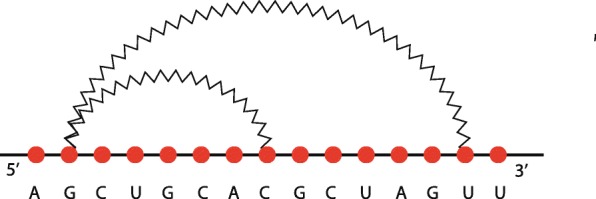
Not admitted RNA structure

As mentioned above, according to the nature of RNA molecules, nesting and crossing are well defined if each nucleotide of the resulting structure forms at most one weak interaction. An example of a not well-defined pseudoloop is depicted in Fig. 9, where the third nucleotide of the primary sequence has two weak interactions. The concatenation operator is not subject to such conditions since the two structures that are attached do not share nucleotides.

It is worth noting that in all the three definitions above it is implicit that the starting pseudoloops *P*_1_ and *P*_2_ are well defined. For the sake of simplicity we did not give a recursive definition to induce the property of being well-defined structurally. The reason is that we will always apply the three operators to hairpin loops (that are indeed well defined) and well-defined combinations of hairpin loops, which yield well-defined pseudoloops by construction.

### A tree grammar for the algebraic RNA expressions

Taking advantage of the three operators defined above, each RNA secondary structure can be defined as a term of a regular tree grammar. Our objective is to generate only trees - or, equivalently, terms - that correspond to valid RNA secondary structures, both pseudoknot-free and pseudoknotted. Moreover, for each secondary structure, the tree (term) must be unique.

Let *B*_∘_={*A*°,*U*°,*G*°,*C*°} and let *B*_∙_={*A*^∙^,*U*^∙^,*G*^∙^,*C*^∙^} be alphabets of RNA base nucleotides bearing the information of being paired or unpaired in a weak interaction. We let $\mathcal {A} = B_{\circ } \cup B_{\bullet } \cup \{(,), \odot, \Cap, \Join \} \cup \{ 2, \dots, K\}$ be the alphabet of our tree grammar, where $K \in \mathbb {N}$ is a constant representing the maximum length of hairpins that we want to consider. For brevity, as introduced in the “Methods” section, we add a lexical level to the grammar allowing strings from $\mathcal {A}^{*}$ in place of single characters. Let *Σ* be the signature defined as follows:

$$\begin{array}{lll} \leftrightarrows & \colon & \mathcal{A}^{*} \times D \times \mathcal{A}^{*} \to D \\ \odot & \colon & D \times \mathcal{A}^{*} \times D \to D \\ \Join & \colon & D \times \mathcal{A}^{*} \times D \to D \\ \Cap & \colon & D \times \mathcal{A}^{*} \times D \to D \\ H & \colon & \mathcal{A}^{*} \to D \end{array} $$

Let $x_{1}, x_{2}, x'_{1}, x'_{2} \in B_{\bullet }, \eta, \eta _{1}, \eta _{2} \in B_{\circ }^{*}$ and $\omega, \omega _{1}, \omega _{2} \in B_{\circ }^{+}$. The regular tree grammar we define for RNA is $\mathcal {G}_{\text {RNA}} = (V, \Sigma, S, \mathcal {P})$, where *V*={*S,T*,*C,N*,*I*} and the set of rewriting rules $\mathcal {P}$ is the following.

$${\begin{aligned} \begin{array}{llll} S & \rightarrow & \leftrightarrows(\eta_{1}, T,\eta_{2}) & \text{ head, main pseudoloop and tail} \\ T & \rightarrow & \odot(T, (\odot, \eta), C) & \text{ concatenation between two pseudoloops} \\ & & \mid C & \text{ no concatenation in current pseudoloop} \\ C & \xrightarrow{c} & \Join \!\!(C, (\Join, k), I) & \text{ crossing between a pseudoloop and an hairpin}\\ & & \mid N & \text{ no crossing in current pseudoloop}\\ N & \xrightarrow{c} & \Cap(T, (\Cap, k), I) & \text{ nesting of a pseudoloop in a hairpin}\\ & & \mid I & \text{current pseudoloop is an hairpin}\\ I & \rightarrow & H(x_{1} \, \omega \, x_{2}) & \text{ hairpin loop}\\ \end{array} \end{aligned}} $$

The rewriting rule for the start symbol *S* formalises that each RNA secondary structure is composed by a head *η*_1_ of unpaired nucleotides, followed by a pseudoloop, followed by a tail *η*_2_ of unpaired nucleotides. Each pseudoloop *T* may be a left-associative concatenation of pseudoloops or just a crossing/nesting/hairpin, by downgrading *T* to *C*, *N* or *I*. Each crossing pseudoloop *C* can be a left-associative sequence of crossings or just a nesting/hairpin, by downgrading *C* to *N* or *I*. Each nesting pseudoloop *N* is composed by a hairpin enclosing a generic embedded pseudoloop *T* or just by a hairpin, by downgrading *N* to *I*.

In the given tree grammar all the nodes corresponding to the concatenation, crossing and nesting operators have a middle child, which is a leaf of the tree, labelled with the operator itself and the additional parameters introduced in Definitions 7, 8 and 9. The presence of these leaf nodes is important because in regular tree grammars the predicate of conditional productions is defined on the yield of the corresponding node. This means that, in order to syntactically check the property of being well-defined, according to the definition of the operators, the predicate *c* in the conditional productions of crossing and nesting must operate on strings. Therefore, these strings, which are the yield of the nodes, must contain the necessary information to recognise the applied operators and their parameters. The definition of *c* is as follows.

Let $u, u', u^{\prime \prime } \in \mathcal {A}^{+}$. The yield of a crossing node or of a nesting node in a derived tree of $\mathcal {G}_{\text {RNA}}$ is a string of the form *u* (*o,k*) *x*_1_ *ω* *x*_2_, where $K \in \mathbb {N}$ and $o \in \{\Join, \Cap \}$, or *x*_1_ *ω*_1_ *x*_2_ (*o,k*) *x*1′ *ω*_2_ *x*2′ if the left pseudoloop reduces to just an hairpin. Let us first define a function $p: \mathcal {A}^{+} \to P$ to transform these yield strings into pseudoloops in the form used in Definitions 7, 8 and 9.

$${\begin{aligned} p(u)= \left\{ \begin{array}{lll} (x_{1}, x_{2}) \langle \omega \rangle & \text{ if} & u = x_{1} \, \omega \, x_{2} \\ p(u') \, o_{k} \, (x_{1}, x_{2}) \langle \omega \rangle & \text{ if} & u = u' \, (o,k) \, x_{1} \, \omega \, x_{2} \text{ and }\ o \in \{\Join, \Cap\}\\ p(u') \, \odot_{\eta} \, p(u^{\prime\prime}) & \text{ if} & u = u' \, (\odot,\eta) \, u^{\prime\prime}\\ \end{array} \right. \end{aligned}} $$

The predicate *c* is defined inductively in accordance with the notion of being well-defined for pseudoloops:

- *c*(*x*_1_ *ω* *x*_2_)=*true*
- *c*(*u*^′^ (⊙,*η*) *u*^′′^)=*c*(*u*^′^)∧*c*(*u*^′′^)
- *c*(*u*^′^ (*o,k*) *x*_1_ *ω* *x*_2_)=*c*(*u*^′^)∧*welldef*(*p*(*u*^′^) *o*_*k*_ (*x*_1_,*x*_2_)〈*ω*〉)

where $o \in \{\Join, \Cap \}$ and *welldef* is a predicate checking whether or not the given application of the operators is well defined according to Definitions 8 and 9.

The particular formulation of the grammar is given with the intention of defining a unique derived tree for each possible secondary structure, pseudoknot-free or pseudoknotted. Let us illustrate this property firstly with an example. We describe, step by step, the unique way to represent the structure in Fig. 6b using the rewriting rules of the grammar. In other words, we introduce a procedure to build a derived tree of grammar $\mathcal {G}_{\text {RNA}}$ starting from a given RNA secondary structure encoded, for instance, as an arc-annotated sequence [47].

**Fig. 10:**
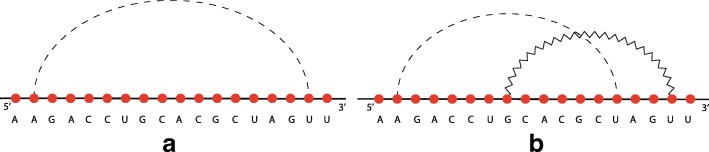
**a**, first and **b**, second step of the procedure for building the derived tree of the structure in Fig. 6**b**

The first step is to recognise the enclosing pseudoloop between the second nucleotide *A*^∙^ and the next to last one *U*^∙^, as illustrated in Fig. 10a. The head *A*° and the tail *U*° of the secondary structure are then immediately identified, resolving uniquely the rewriting rule for the start symbol *S*. Now we have to decompose the just identified pseudoloop rewriting the non-terminal symbol *T*. The first possible decomposition to consider is the simplest one, i.e., concatenation. In this case the pseudoloop cannot be decomposed into sequences of concatenated pseudoloops. Thus, the rewriting rule *T*→*C* is applied and the pseudoloop must be decomposed into a crossing or into a nesting. The way to deterministically decide this, according to the structure of the grammar, is to select the hairpin inside the pseudoloop that has the rightmost paired nucleotide. In our case such hairpin is *α*_1_=*G*^∙^*C*°*A*°*C*°*G*°*C*°*U*°*A*°*G*°*U*^∙^. Then, it is checked if it crosses with some other arc inside the pseudoloop. This is so in our case, thus the grammar rewriting rule to be selected is the one for crossing, *C*→⋊⋉(*C*,(⋊⋉,*k*),*I*), where *I* is rewritten with *I*→*H*(*α*_1_). It follows that *C* must be the pseudoloop that results by eliminating the hairpin *α*_1_ from the originally identified pseudoloop, which yields the one starting at the second nucleotide of the sequence and ending at the fourteenth, depicted with a dashed line in Fig. 10b. The value of *k* follows as well: it is the position at which the left paired nucleotide of *α*_1_,*G*^∙^, is in the new identified pseudoloop, i.e., 7. At this point we identified the rewriting rule to be used and all its components apart from *C*, which must be recursively analysed.

**Fig. 11:**
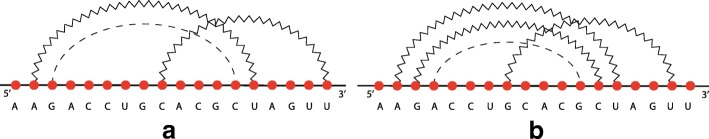
**a**, third and **b**, fourth step of the procedure for building the derived tree of the structure in Fig. 6b

Let us then consider the pseudoloop in Fig. 10b. Again, this pseudoloop cannot be decomposed as a sequence of concatenations, so we re-apply the same technique and consider the hairpin that has the rightmost paired nucleotide in the pseudoloop. This is *α*_2_=*A*^∙^*G*°*A*°*C*°*C*°*U*°*G*°*C*°*A*°*C*°*G*°*C*°*U*^∙^, which does not cross with any arc in the pseudoloop. Therefore the rewriting rule to be used first is *C*→*N* and then the one to decompose the pseudoloop as a nesting, $ N \rightarrow \Cap (T, (\Cap, k), I)$, where *I* is rewritten with *I*→*H*(*α*_2_). By eliminating *α*_2_ from the pseudoloop we are left with the pseudoloop in the interior part of the nesting, i.e., the one from the third nucleotide, *G*^∙^, and the thirteenth one, *C*^∙^, depicted as a dashed line in Fig. 11a. In this case it follows that *k*=2.

**Fig. 12:**
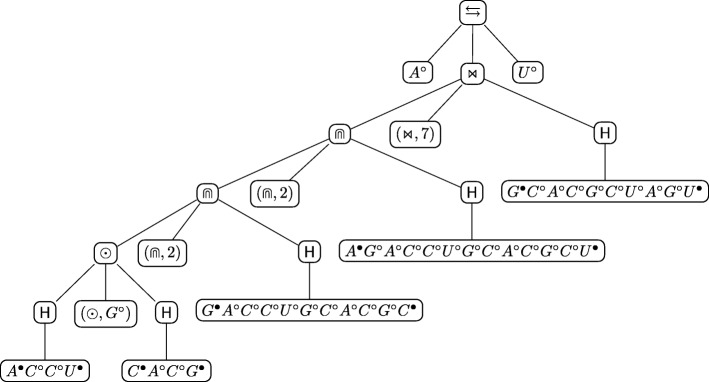
Algebraic RNA tree of the structure in Fig. 6b according to the regular tree grammar $\mathcal {G}_{\text {RNA}}$

The next step is to recursively decompose the last identified pseudoloop. It is easy to see that it is again a nesting with *k* equal to 2. The resulting pseudoloop to be further decomposed is the one depicted with a dashed line in Fig. 11b. This particular pseudoloop can indeed be decomposed as a concatenation of two pseudoloops, which are actually two hairpins: *A*^∙^*C*°*C*°*U*^∙^ and *C*^∙^*A*°*C*°*G*^∙^. The value of *η* for the concatenation is *G*°. Since there are no more non-hairpin pseudoloops to decompose, the procedure ends. The unique *derived tree* of the grammar associated to the structure of Fig. 6b, built using the procedure illustrated above, is shown in Fig. 12.

#### **Theorem 1**

Let $\mathcal {S}$ be an RNA secondary structure. Then, there is only one derived tree of the tree grammar $\mathcal {G}_{\text {RNA}}$ associated to $\mathcal {S}$.

#### *Proof*

Given any $\mathcal {S}$, it is possible to follow a procedure analogous to the one illustrated for the structure depicted in Fig. 6b. Firstly, by hypothesis, $\mathcal {S}$ respects the constraint on weak interactions of RNA, i.e., there are not nucleotides that have more than one weak interaction. This means that the application of the operators at each step is well-defined. Secondly, the choice of the operator to use at each step of the procedure is uniquely determined by the structure of $\mathcal {S}$. Finally, in case of crossing and nesting, the choice of the hairpin to identify as the one to remove from the pseudoloop is unique as well. Thus, the way to construct the derived tree for $\mathcal {S}$ is unique. □

#### **Corollary 1**

Any secondary structure can be uniquely decomposed in terms of hairpins together with a head and a tail.

#### *Proof*

Given any structure $\mathcal {S}$, the derived tree of the grammar $\mathcal {G}_{\text {RNA}}$ associated to $\mathcal {S}$, as determined in the proof of Theorem 1, expresses the structure as a unique combination of hairpins using the operators for concatenation, crossing and nesting. The head and the tail are attached at the beginning and at the end. □

As an example, consider the derived tree in Fig. 12, associated to the structure of Fig. 6b. Let *α*_3_=*G*^∙^*A*°*C*°*C*°*U*°*G*°*C*°*A*°*C*°*G*°*C*^∙^,*α*_4_=*C*^∙^*A*°*C*°*G*^∙^ and *α*_5_=*A*^∙^*C*°*C*°*U*^∙^ be sequences of nucleotides representing hairpins and let *α*_1_ and *α*_2_ be as above. The decomposition in hairpins expressed by the derived tree is the yield of the tree itself:

$$A^{\circ} \, [[[[\alpha_{5} \, (\odot, G^{\circ}) \,\alpha_{4}] \, \!(\Cap, 2) \, \alpha_{3}] \, \!(\Cap, 2) \, \alpha_{2}] \, \!(\Join, 7) \, \alpha_{1}] \, \!U^{\circ} $$

where square brackets are used to emphasise the structure of the string induced by the tree, but are not part of the string.

#### **Theorem 2**

Let *t* be a derived tree of the regular tree grammar $\mathcal {G}_{\text {RNA}}$. Then, the structure of *t* corresponds to an RNA secondary structure.

#### *Proof*

By definition of derived tree for a regular tree grammar, the predicate *c* of conditional productions for crossing and nesting nodes are all satisfied. This means that the constraints on the weak interactions are satisfied and that the pseudoloops that are identified in *t* are all well defined. To see that then *t* represents indeed a secondary structure it is sufficient to observe that each time in *t* a conditional production is applied, one hairpin is added to the structure in a well-defined way. Moreover, each time in *t* the rewriting rule *T*→⊙(*T*,(⊙,*η*),*C*) is applied, several well-defined substructures are just concatenated, forming well-defined structures by definition because concatenation is alway well defined. □

Finally, we can characterise the property of a structure of being pseudoknot-free or pseudoknotted by looking at the operators that are needed to represent it.

#### **Theorem 3**

Let $\mathcal {S}$ be a secondary structure. $\mathcal {S}$ is pseudoknotted if and only if the derived tree associated to $\mathcal {S}$ by the regular tree grammar $\mathcal {G}_{\text {RNA}}$ contains at least an internal node labelled with ⋊⋉. Otherwise, $\mathcal {S}$ is pseudoknot-free.

#### *Proof*

It is sufficient to observe that in the process of constructing the derivation tree associated to $\mathcal {S}$, as in the proof of Theorem 1, the internal node ⋊⋉, corresponding to a crossing, is selected only if the current rightmost hairpin is actually crossing with some other hairpin of the pseudoloop on the left. Thus, if the rule is not used in the whole tree then there is no hairpin crossing with another in the structure, i.e., the structure is pseudoknot-free. □

In other words, we can characterise all pseudoknot-free structures using only the concatenation and the nesting operator. Crossing is needed only for pseudoknotted structures. In order to emphasise the algebraic nature of the tree we introduce the following name.

#### **Definition 10**

(Algebraic RNA Tree) Let $\mathcal {S}$ be a secondary structure. The *Algebraic RNA Tree* of $\mathcal {S}$ is defined as the unique derived tree of the grammar $\mathcal {G}_{\text {RNA}}$ associated to $\mathcal {S}$.

### Algebraic structural pseudoknot RNA alignment

Our first application of algebraic RNA trees introduced so far is in the field of structure comparison. In particular, we are interested in comparing RNA secondary structures *structurally*, i.e., looking at the (possibly pseudoknotted) structures by neglecting the kind of base pairs that created the weak interactions, i.e., without depending on the primary structure. For this purpose, algebraic RNA trees contain unnecessary information that can be abstracted, i.e., the identity of the nucleotides forming the hairpins. Moreover, in algebraic RNA trees the positions at which the pseudoloops are connected are expressed by the parameters *k* of the crossing and the nesting operators. According to the nature of the algebraic operators, such positions are *relative* to the involved operands. This is not convenient for a structural comparison of the whole structure, for which it is necessary to reconstruct the corresponding *absolute* positions inside the primary sequence. Therefore, we make a further step by introducing *structural RNA trees* as an abstraction of algebraic RNA trees in which the information about the identity of the nucleotides is forgotten and the absolute positions of the involved hairpins are reported.

#### **Definition 11**

(Structural RNA Tree) A *structural RNA tree* is an ordered labelled tree such that:

- each interior node has two children and is labelled with $\odot, \Cap $ or with an element of $\{(\Join, h) \mid h \in \mathbb {N} \}$;
- each leaf is labelled with *H*(start,stop), where $\text {start},\text {stop} \in \mathbb {N}$.

**Fig. 13:**
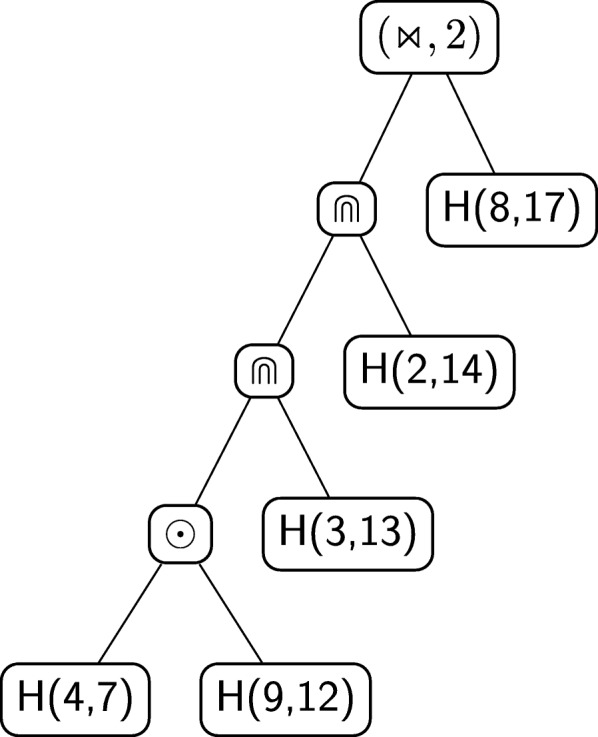
The structural RNA tree corresponding to the algebraic RNA tree in Fig. 12

Figure 13 shows a structural RNA tree that is the abstraction of the algebraic RNA tree of Fig. 12, which in turn corresponds to the structure of Fig. 6b. First, note that the original root of the algebraic RNA tree, labelled with $\leftrightarrows $, is eliminated, together with the head and the tail sequences. The other internal nodes labelled with operators in the algebraic RNA tree remain the same in the structural RNA tree, apart from the crossing operator, which is paired with a number *h*. This *h* is the number of crossing interactions of the current hairpin and will be explained later in more detail. The middle child of each internal node labelled with an operator of the algebraic RNA tree disappears in the structural RNA tree and the right child becomes a leaf node *H*(start,stop) representing the same hairpin loop of the algebraic RNA tree, but showing the absolute positions at which the hairpin starts and stops in the original structure.

**Fig. 14:**
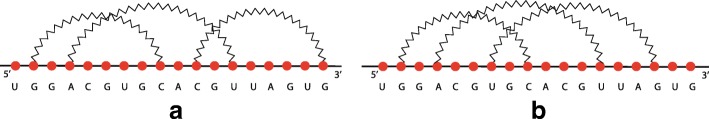
Two different RNA secondary structures having the same pattern of application of the crossing operator. In **a**, the rightmost weak interaction crosses only with one of the other two. In **b**, the rightmost weak interaction crosses with both the other two

**Fig. 15:**
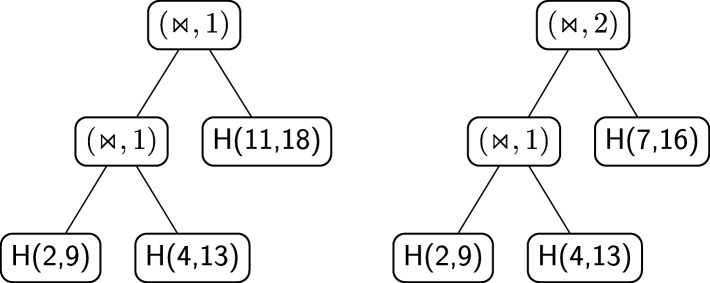
On the left, the structural RNA tree of the structure shown in Fig. 14a. On the right, the structural RNA tree of the structure shown in Fig. 14b

A structural RNA tree can be obtained from an algebraic RNA tree through a depth-first visit of the latter in which, for each internal node *ν* labelled with an operator, the list of hairpins (with absolute starting and stopping positions) constituting the pseudoloop associated to the left child is returned from the recursive function. Using this list, it is possible to calculate, whenever the current operator of *ν* is a crossing, the number of hairpins in the pseudoloop of the left child that actually cross with the hairpin on the right child. Given two hairpins *H*(*i,j*) and *H*(*i*^′^,*j*^′^) such that *j*^′^<*j*, we say that *H*(*i,j*) crosses with *H*(*i*^′^,*j*^′^) if and only if *i*^′^<*i*<*j*^′^. Consider the structure depicted in Fig. 14a and the relative structural RNA tree shown in the left part of Fig. 15. The root is labelled with (⋊⋉,1) because the hairpin *H*(11,18) crosses only with the hairpin *H*(4,13) and not with *H*(2,9), which are the two hairpins constituting the pseudoloop associated to the left child of the root. Differently, if we consider the structure depicted in Fig. 14b and the relative structural RNA tree shown in the right part of Fig. 15, the root is labelled with (⋊⋉,2). Indeed, in this case the hairpin *H*(7,16) of the right child of the root crosses with both the hairpins *H*(2,9) and *H*(4,13) of the pseudoloop associated with the left child.

**Fig. 16:**
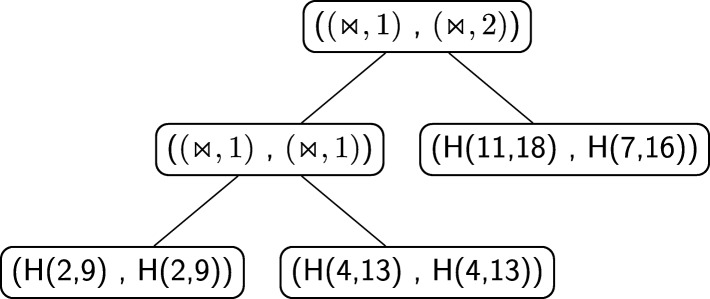
Alignment of the structural RNA trees of Fig. 15

Figure 16 shows an alignment tree of the two structural RNA trees of Fig. 15. Let us suppose, as we will explain better later, that the score of aligning two hairpins *H*(*i,j*) and *H*(*i*^′^,*j*^′^) such that *i*≠*i*^′^ or *j*≠*j*^′^ is zero. If the nodes containing the crossing operator were without the number of crossing interactions *h*, the alignment tree of Fig. 16 would have been indeed an optimal one, with tree alignment distance 0. Thus, the two structures of Fig. 14 would have been considered equal. This is not correct for the measure of comparison we want to define because indeed the two structures are different from a *structural* point of view. This issue is the reason why the number *h* has been introduced in the crossing nodes of structural RNA trees. In this way a positive score *c*_*m*_ can be fixed and, when aligning two nodes (⋊⋉,*h*) and (⋊⋉,*h*^′^), a score *c*_*m*_·|*h*−*h*^′^| can be assigned to the pair. Using this score, the distance between the two structures of Fig. 14 becomes *c*_*m*_·|1−2|+*c*_*m*_·|1−1|=*c*_*m*_·1+0=*c*_*m*_>0, i.e. the structures are considered different.

Suppose we are given two RNA secondary structures and their algebraic RNA trees obtained by the grammar $\mathcal {G}_{RNA}$. To compare the two structures, we align the corresponding structural RNA trees. We define the scoring function *σ*_s_ as follows:

$${\begin{aligned} \begin{array}{ll} \sigma_{\mathsf{s}}((\Join, h), (\Join, h')) = c_{m} \cdot |h - h'| & \text{ crossing mismatch}\\ \sigma_{\mathsf{s}}(op_{1}, op_{2}) = o_{r} & \text{ replace operator}\\ \sigma_{\mathsf{s}}(op, -) = \sigma_{\mathsf{s}}(-, op) = o_{\text{di}} & \text{ delete or insert operator}\\ \sigma_{\mathsf{s}}(H(i, j), H(i', j')) = 0 & \text{ replace hairpin with hairpin}\\ \sigma_{\mathsf{s}}(H(i, j), op) = \sigma_{\mathsf{s}}(op, H(i, j)) = +\infty & \text{ replace hairpin with operator}\\ \sigma_{\mathsf{s}}(H(i, j), -) = \sigma_{\mathsf{s}}(-, H(i, j)) = h_{\text{di}} & \text{ delete or insert hairpin}\\ \end{array} \end{aligned}} $$

where $h, h', i, i', j, j' \in \mathbb {N}, op, op_{1}, op_{2} \in \{(\Join, n), \Cap, \odot \}$ and $c_{m}, o_{r}, o_{\text {di}}, h_{\text {di}} \in \mathbb {R}$ are score constants.

The scoring function *σ*_s_ is quite standard regarding the pairs containing an insertion or a deletion of an operator or of an hairpin. Concerning the pairs with a replacement, we already discussed the case of two crossing operators with a possibly different number *h*. The case in which an hairpin is replaced with an hairpin with possibly different absolute position is assigned score zero. The reason for this is again the fact that we want the measure of comparison independent from the primary sequence. The structural interplay among the hairpins is structurally expressed by the operators and the structural differences between crossing hairpins is already considered by the case of two crossing operators. Therefore, assigning a positive score to differences in the absolute positions of two aligned hairpins would introduce a dependence from the primary sequence and would be useless for our purposes. Finally, the replacement of an operator with an hairpin should always be avoided because the resulting alignment tree would not conserve the shape reflecting the application of the operators to hairpins and sub-terms. This is the reason why the score assigned to such a replacement is infinite. We can now define the Algebraic Structural Pseudoknot RNA Alignment (ASPRA) distance.

#### **Definition 12**

(ASPRA Distance) Let $\mathcal {S}_{1}$ and $\mathcal {S}_{2}$ be two RNA secondary structures with or without pseudoknots and let *t*_1_ and *t*_2_ be the structural RNA trees corresponding to their algebraic RNA trees. The *Algebraic Structural Pseudoknot RNA Alignment (ASPRA) distance* between $\mathcal {S}_{1}$ and $\mathcal {S}_{2}$, denoted by $d_{\text {aspra}}(\mathcal {S}_{1}, \mathcal {S}_{2})$, is defined as follows:

$$d_{\text{aspra}}(\mathcal{S}_{1}, \mathcal{S}_{2}) = \min \{ \sigma_{\mathsf{s}}(L) \mid L \text{ is an alignment of}\ t_{1} \text{ and}\ t_{2} \} $$

**Fig. 17:**
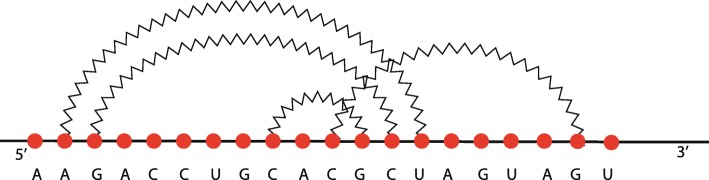
An RNA secondary structure to be compared with the one in Fig. 6b

**Fig. 18:**
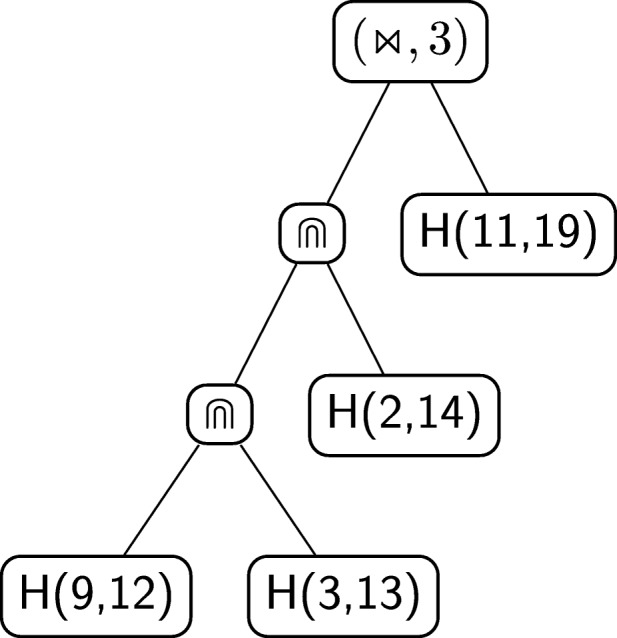
The structural RNA tree of the structure in Fig. 17

**Fig. 19:**
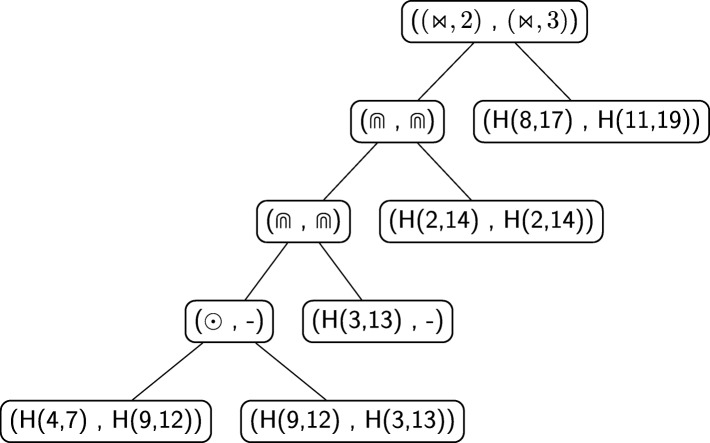
One of the optimal alignments of the structural RNA trees in Figs. 13 and 18

Let us give an example by calculating the distance between the RNA secondary structure introduced in Fig. 6b, say $\mathcal {S}_{1}$, and the RNA secondary structure illustrated in Fig. 17, say $\mathcal {S}_{2}$. The structural RNA tree of the structure $\mathcal {S}_{2}$ is shown in Fig. 18. Figure 19 shows an optimal alignment tree of the structural RNA trees of $\mathcal {S}_{1}$ and $\mathcal {S}_{2}$. The distance is

$${\begin{aligned} d_{\text{aspra}}(\mathcal{S}_{1}, \mathcal{S}_{2}) \,=\, \sigma_{\mathsf{s}}((\bowtie,2),(\bowtie,3)) \,+\, \sigma_{\mathsf{s}}(\odot,-) \,+\, \sigma_{\mathsf{s}}(H(3,13),-) \,=\, c_{m} + o_{\text{di}} + h_{\text{di}} \end{aligned}} $$

having all the other pairs score 0.

To conclude this section, it is worth noting that the unit score *c*_*m*_ to be assigned to a mismatch in the number of crossing interactions of two aligned crossing nodes (⋊⋉,*h*),(⋊⋉,*h*^′^) should have a different scale with respect to the other scores. This would reflect the fact that the two nodes actually have the same operator and the difference that we measure is *local* to these operators. The replacement of an operator with another one, instead, is a proper replacement in the classical sense of edit operations. Therefore, the score constants should be chosen such that *c*_*m*_≪*o*_*r*_,*o*_id_,*h*_id_. Possible choices are *o*_*r*_=*o*_id_=*o*_id_=1,*c*_*m*_=1/100 or, to avoid rounding errors, *o*_*r*_=*o*_id_=*o*_id_=100,*c*_*m*_=1.

## Discussion

We have introduced a new algebraic representation for RNA secondary structures with arbitrary pseudoknots. We have called it *algebraic RNA tree*, which is derived from a regular tree grammar using three main operators: *concatenation*, *nesting* and *crossing*. The language of terms generated by the given tree grammar is shown to be in one-to-one correspondence with the set of RNA secondary structures with arbitrary pseudoknots. As a first application of algebraic RNA trees we have defined a measure of comparison, called *ASPRA distance*, that is able to compare RNA secondary structures with arbitrary pseudoknots *structurally*, i.e., without depending on the primary structure. The distance is obtained by using the classical tree alignment algorithm on *structural RNA trees*, abstractions of algebraic RNA trees. In this section we give the necessary information to practically use the notions introduced so far on real RNA molecules.

The procedures to construct algebraic and structural RNA trees and to calculate the ASPRA distance have been implemented in the ASPRAlign Java application [42]. The source code of ASPRAlign, together with documentation and examples, is publicly available under the GNU General Public Licence, version 3.

ASPRAlign accepts as input RNA secondary structures in the Extended Dot-Bracket Notation format [48], typically supported by public database of structures, or in the Arc Annotated Sequence format. The latter format is derived from the former by substituting the dot-bracket string with a list of weak interactions expressed as pairs (*i*_1_,*j*_1_);(*i*_2_,*j*_2_);…;(*i*_*m*_,*j*_*m*_) where each index *i*_*k*_,*j*_*k*_ belongs to the interval [1,*n*] (*n* being the length of the primary sequence) and *i*_*k*_<*j*_*k*_+1 for all *k*. The weak interactions can be given in any order and the indices *i*_*k*_,*j*_*k*_ are the starting and the stopping positions of the *k*-th weak interaction. The default output format of ASPRAlign for trees is a string resulting form a depth-first visit of the given tree following the format (~node-label~, [list-of-children]). An alternative output format for a tree is LATE X code to obtain a graphical tree representation like the ones in Figs. 12, 13 and 16.

Starting from a secondary structure given in arc annotated sequence format, as specified above, ASPRAlign builds the algebraic RNA tree in time

$$\mathcal{O}\left(n + \sum_{k=1}^{m} (j_{k} - i_{k})\right) $$

where (*j*_*k*_−*i*_*k*_) is the length of the *k*-th weak interaction. The construction of an arc annotated sequence starting from an extended dot-bracket notation format can be done in $\mathcal {O}(n)$. The building of the structural RNA tree is currently implemented from scratch, i.e. starting from the secondary structure specification, with the same time complexity of building the algebraic RNA tree. Alternatively, the structural RNA tree can be derived from a visit of the algebraic RNA tree as discussed in the previous subsection.

ASPRAlign uses the implementation of the Jiang et al. algorithm [20] for tree alignment provided by the Java package fr.orsay.lri.varna.models.treealign of the StatAling software package [49, 50]. The time complexity of the Jiang et al. algorithm is $\mathcal {O}(|t_{1}|\cdot |t_{2}|\cdot (\text {deg}(t_{1}) + \text {deg}(t_{2}))^{2})$ where |*t*| is the number of nodes of the tree *t* and deg(*t*) is the degree of the tree *t*, i.e. the maximum number or children of any node in the tree. In the case of ASPRAlign the trees *t*_1_ and *t*_2_ are the structural RNA trees of the given structures. A structural RNA tree has always degree 2 and the number of nodes is linear in the number of weak interactions. Thus, the tree alignment and the computation of the ASPRA distance of two secondary structures with *m*_1_ and *m*_2_ weak interactions is performed in time $\mathcal {O}(m_{1} \cdot m_{2})$.

## Conclusions

In this paper we have introduced algebraic RNA trees and structural RNA trees to represent uniquely RNA secondary structures with arbitrary pseudoknots. This has been achieved by representing the structures as expressions of an algebraic language with three operators and simple hairpin loops as operands. While in classical representations pseudoknotted structures can not be represented by a tree, this is quite natural using our operators. Based on structural RNA trees, we have also defined the ASPRA distance to compare RNA secondary structures without taking into account the primary sequences and focusing mainly on the motifs of the structures. Such a measure of comparison is useful because the secondary structure is more preserved than the primary one during evolution. Our distance has the advantage to consider all the weak interactions including the pseudoknots, while in other measures of comparison present in the literature only subclasses of pseudoknots are considered. We have implemented the procedures to build the trees and to compute the distance in an open source Java application called ASPRAlign [42].

As an immediate continuation of the present work, the ASPRA distance will be tested on real RNA secondary structure with pseudoknots that are available in public repositories such as the Worldwide Protein Data Bank Database [51] and the Pseudobase++ Database [52]. This work will be carried out in collaboration with experts of the biological domain in order to test both the usability of the software and the impact of our new measure of comparison on the creation of new biological knowledge.

On the theoretical side, the natural extension of our algebraic approach is the formalisation of the three operators in an algebraic structure with a proper axiomatisation. This would allow us to study the properties of the RNA secondary structures with arbitrary pseudoknots in a compositional way.

Beyond the structural comparison based on the ASPRA distance we aim at applying our approach to the folding problem of RNA secondary structures with pseudoknots. To this end, the Algebraic Dynamic Programming framework, together with the tree language already introduced in this paper, is a good starting point. The objective is to derive an algorithm that works for arbitrary pseudoknots, instead of classes of pseudoknots, as it is in the current state of the art. Moreover, an improvement of the efficiency of the existing algorithms for the various classes of pseudoknots might be reached by exploiting suitable properties of our operators. The same problem can be faced using the algebraic representation together with learning algorithms and adaptability checking typical of complex systems [53–55].

Finally, based on our preliminary results [56, 57] and using our algebraic approach, we plan also to deal with the problem of RNA classification.

## Abbreviations

A: Adenine
ADP: Algebraic Dynamic Programming
ASPRA distance: Algebraic Structural Pseudoknot RNA Alignment distance
C: Cytosine
G: Guanine
U: Uracil

## References

[1] Waterman MS (1978). Secondary Structure of Single-Stranded Nucleic Acids. Studies on Foundations and Combinatorics, Advances in Mathematics Supplementary Studies, vol. 1.

[2] Waterman MS, Smith TF (1978). RNA secondary structure: a complete mathematical analysis. Math Biosci.

[3] Dam ET, Pleij K, Draper D (1992). Structural and functional aspects of RNA pseudoknots. Biochemistry.

[4] Staple DW, Butcher SE (2005). Pseudoknots: RNA Structures with Diverse Functions. PLoS Biol.

[5] Rastogi T, Beattie TL, Olive JE, Collins RA (1996). A long-range pseudoknot is required for activity of the Neurospora VS ribozyme. EMBO J.

[6] Ke A, Zhou K, Ding F, Cate JH, Doudna JA (2004). A conformational switch controls hepatitis delta virus ribozyme catalysis. Nature.

[7] Shen LX, Tinoco Jr I (1995). The structure of an RNA pseudoknot that causes efficient frameshifting in mouse mammary tumor virus. J Mol Biol.

[8] Egli M, Minasov G, Su L, Rich A (2002). Metal ions and flexibility in a viral RNA pseudoknot at atomic resolution. Proc Natl Acad Sci.

[9] Hofacker IL, Fekete M, Flamm C, Huynen MA, Rauscher S, Stolorz PE, Stadler PF (1998). Automatic detection of conserved RNA structure elements in complete RNA virus genomes. Nucleic Acids Res.

[10] Caetano-Anollés G (2002). Tracing the evolution of RNA structure in ribosomes. Nucleic Acids Res.

[11] Wang H-Y, Lee S-C (2002). Secondary Structure of Mitochondrial 12S rRNA Among Fish and Its Phylogenetic Applications. Mol Biol Evol.

[12] Wuyts J, De Rijk P, Van de Peer Y, Pison G, Rousseeuw P, De Wachter R (2000). Comparative analysis of more than 3000 sequences reveals the existence of two pseudoknots in area V4 of eukaryotic small subunit ribosomal RNA. Nucleic Acids Res.

[13] Chai W, Stewart V (1999). RNA sequence requirements for NasR-mediated, nitrate-responsive transcription antitermination of the Klebsiella oxytoca M5al nasF operon leader. J Mol Biol.

[14] Höchsmann M, Voss B, Giegerich R (2004). Pure Multiple RNA Secondary Structure Alignments: A Progressive Profile Approach. IEEE/ACM Trans Comput Biol Bioinforma.

[15] Shapiro BA, Zhang K (1990). Comparing multiple RNA secondary structures using tree comparisons. Bioinformatics.

[16] Corpet F, Michot B (1994). RNAlign program: alignment of RNA sequences using both primary and secondary structures. Bioinformatics.

[17] Jiang T, Lin G, Ma B, Zhang K (2002). A General Edit Distance between RNA Structures. J Comput Biol.

[18] Selkow SM (1977). The tree-to-tree editing problem. Inf Process Lett.

[19] Tai K-C (1979). The Tree-to-Tree Correction Problem. J ACM.

[20] Jiang T, Wang L, Zhang K (1995). Alignment of trees - an alternative to tree edit. Theor Comput Sci.

[21] Höchsmann M, Töller T, Giegerich R, Kurtz S (2003). Local similarity in RNA secondary structures. Computational Systems Bioinformatics. CSB2003. Proceedings of the 2003 IEEE Bioinformatics Conference.

[22] Lorenz R, Bernhart SH, Höner zu Siederdissen C, Tafer H, Flamm C, Stadler PF, Hofacker IL. ViennaRNA Package 2.0. Algoritm Mol Biol. 2011;6(26). https://almob.biomedcentral.com/articles/10.1186/1748-7188-6-26. 10.1186/1748-7188-6-26 PMC3319429 22115189

[23] Chauve C, Courtiel J, Ponty Y. An Unambiguous And Complete Dynamic Programming Algorithm For Tree Alignment. Submitted. Version 1. 2015. https://hal.inria.fr/hal-01154030. Accessed 28 Sep 2018.

[24] Schirmer S, Giegerich R, Giancarlo R, Manzini G (2011). Forest Alignment with Affine Gaps and Anchors. Combinatorial Pattern Matching. CPM 2011. Lecture Notes in Computer Science, vol. 6661.

[25] Bille P (2005). A survey on tree edit distance and related problems. Theor Comput Sci.

[26] Harrison MA (1978). Introduction to Formal Language Theory.

[27] Möhl M, Will S, Backofen R (2010). Lifting Prediction to Alignment of RNA Pseudoknots. J Comput Biol.

[28] Han B, Dost B, Bafna V, Zhang S (2008). Structural Alignment of Pseudoknotted RNA. J Comput Biol.

[29] Yoon B-J (2009). Efficient alignment of RNAs with pseudoknots using sequence alignment constraints. EURASIP J Bioinforma Syst Biol.

[30] Wong TKF, Wan K-L, Hsu B-Y, Cheung BWY, Hon W-K, Lam T-W, Yiu S-M (2011). RNASAlign: RNA Structural Alignment System. Bioinformatics.

[31] Huang Z, Wu Y, Robertson J, Feng L, Malmberg RL, Cai L (2008). Fast and accurate search for non-coding RNA pseudoknot structures in genomes. Bioinformatics.

[32] Fallmann J, Will SS, Engelhardt J, Grüning B, Backofen R, Stadler PF (2017). Recent advances in RNA folding. J Biotechnol.

[33] Akutsu T (2000). Dynamic programming algorithms for RNA secondary structure prediction with pseudoknots. Discret Appl Math.

[34] Nebel MEME, Weinberg F (2012). Algebraic and Combinatorial Properties of Common RNA Pseudoknot Classes with Applications. J Comput Biol.

[35] Reeder J, Giegerich R (2004). Design, implementation and evaluation of a practical pseudoknot folding algorithm based on thermodynamics. BMC Bioinformatics.

[36] Giegerich R, Meyer C, Kirchner H, Ringeissen C (2002). Algebraic Dynamic Programming. Algebraic Methodology and Software Technology. AMAST 2002. Lecture Notes in Computer Science, vol. 2422.

[37] Giegerich R, Meyer C, Steffen P (2004). A discipline of dynamic programming over sequence data. Sci Comput Program.

[38] Berkemer SJ, Höner zu Siederdissen C, Stadler PF (2017). Algebraic Dynamic Programming on Trees. Algorithms.

[39] Riechert M, Höner zu Siederdissen C, Stadler PF (2016). Algebraic dynamic programming for multiple context-free grammars. Theor Comput Sci.

[40] Ponty Y, Saule C, Przytycka TM, Sagot MF (2011). A Combinatorial Framework for Designing (Pseudoknotted) RNA Algorithms. Algorithms in Bioinformatics. WABI 2011. Lecture Notes in Computer Science, vol. 6833.

[41] Allen JF (1983). Maintaining knowledge about temporal intervals. Commun ACM.

[42] Quadrini M, Tesei L, Merelli E. ASPRAlign - Algebraic Structural Pseudoknot RNA Alignment. 2018. https://github.com/bdslab/aspralign. Accessed 28 Sep 2018.

[43] Thatcher JW (1976). Characterizing derivation trees of context-free grammars through a generalization of finite automata theory. J Comput Syst Sci.

[44] Gécseg F, Steinby M (1997). Tree Languages. Handbook of Formal Languages.

[45] Giegerich R, Steffen P, Boiten EA, Möller B (2002). Implementing Algebraic Dynamic Programming in the Functional and the Imperative Programming Paradigm. Mathematics of Program Construction. MPC 2002. Lecture Notes in Computer Science, vol. 2386.

[46] Schirmer S, Ponty Y, Giegerich R, Gorodkin J, Ruzzo W (2014). Introduction to RNA Secondary Structure Comparison. RNA Sequence, Structure, and Function: Computational and Bioinformatic Methods. Methods in Molecular Biology (Methods and Protocols), vol. 1097.

[47] Blin G, Touzet H, Crestani F, Ferragina P, Sanderson M (2006). How to Compare Arc-Annotated Sequences: The Alignment Hierarchy. String Processing and Information Retrieval. SPIRE 2006. Lecture Notes in Computer Science.

[48] ViennaRNA Package 2.0. RNAlib-2.4.9 Documentation. 2018. https://www.tbi.univie.ac.at/RNA/ViennaRNA/doc/html/rna_structure_notations.html. Accessed 28 Sep 2018.

[49] StatAlign v3.2. An Extendable Software Package for Joint Bayesian Estimation of Alignments and Evolutionary Trees. 2018. https://statalign.github.io. Accessed 28 Sep 2018. 10.1093/bioinformatics/btn457 18753153

[50] Arunapuram P, Edvardsson I, Golden M, Anderson JWJ, Novàk A, Sükösd Z, Hein J (2013). StatAlign 2.0: combining statistical alignment with RNA secondary structure prediction. Bioinformatics.

[51] Berman Helen, Henrick Kim, Nakamura Haruki (2003). Announcing the worldwide Protein Data Bank. Nature Structural & Molecular Biology.

[52] Taufer M, Licon A, Araiza R, Mireles D, van Batenburg FHD, Gultyaev AP, Leung M-Y (2009). PseudoBase++: an extension of PseudoBase for easy searching, formatting and visualization of pseudoknots. Nucleic Acids Res.

[53] Merelli E, Pettini M, Rasetti M (2015). Topology driven modeling: the IS metaphor. Nat Comput.

[54] Merelli E, Paoletti N, Tesei L (2016). Adaptability checking in complex systems. Sci Comput Program.

[55] Mamuye AL, Merelli E, Tesei L (2016). A Graph Grammar for Modelling RNA Folding. Electron Proc Theor Comput Sci EPTCS.

[56] Quadrini M, Culmone R, Merelli E, Martín-Vide C, Neruda R, Vega-Rodríguez M (2017). Topological Classification of RNA Structures via Intersection Graph. Theory and Practice of Natural Computing. TPNC 2017. Lecture Notes in Computer Science, vol. 10687.

[57] Quadrini M, Merelli E (2018). Loop-loop Interaction Metrics on RNA Secondary Structures with Pseudoknots. Proceedings of the 11th International Joint Conference on Biomedical Engineering Systems and Technologies - Volume 4: BIOINFORMATICS.

